# Neglected Comminuted Fracture of the Capitellum

**DOI:** 10.7759/cureus.7765

**Published:** 2020-04-21

**Authors:** Apoorv Sehgal, Pratyush Shahi, Kuldeep Bansal, Ahmer Zafar, Siddharth Pathak

**Affiliations:** 1 Orthopaedics, University College of Medical Science and Guru Teg Bahadur Hospital, Delhi, IND; 2 Orthopaedics, University College of Medicine Science and Guru Teg Bahadur Hospital, Delhi, IND

**Keywords:** capitellum, comminuted fracture, neglected, type 4, herbert screw, elbow anterolateral approach

## Abstract

Fracture of the capitellum is a rare injury, accounting for about 1% of the fractures around the elbow. We report the case of a young adult with elbow pain and swelling presenting to us three weeks after the injury. Radiographs suggested a comminuted fracture of the capitellum extending medially to the trochlea. Using the anterolateral approach to the elbow, an open reduction and internal fixation of the fracture with screws was done. The procedure had an excellent functional outcome. Through this case report, we aim to highlight the importance of radiographic assessment and decision-making regarding the surgical approach and choice of the implant in the treatment of comminuted capitellar fractures.

## Introduction

Fracture of the capitellum is a rare injury, accounting for about 1% of the fractures around the elbow [1]. It results from low-energy trauma, usually a fall on an outstretched hand. Comminuted capitellar fractures are particularly difficult to manage. Although there are a number of classification systems described for this fracture, the level of comminution is accounted for only in the one proposed by Dubberley et al. [2]. This case report aims to highlight the importance of radiographic assessment and decision-making regarding the surgical approach and choice of the implant in the treatment of comminuted capitellar fractures. 

## Case presentation

A 24-year-old male came to us with pain and swelling in the right elbow. He had a history of fall on the outstretched hand three weeks back. He had taken treatment from a local osteopath in the form of crepe bandaging, but his symptoms did not subside. On examination, there was a generalized swelling of the right elbow along with tenderness over the lateral condyle of the humerus. There was no distal neurovascular deficit. 

**Figure 1:**
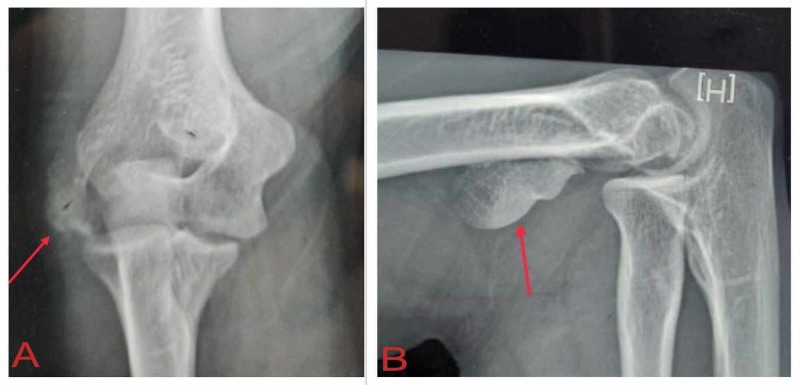
Preoperative X-rays of the right elbow. The arrows show the fractured capitellar fragment in the (A) anteroposterior and (B) lateral views.

The anteroposterior and lateral view X-rays showed a coronal fracture of the lateral column of the distal humerus (Figure 1).

**Figure 2:**
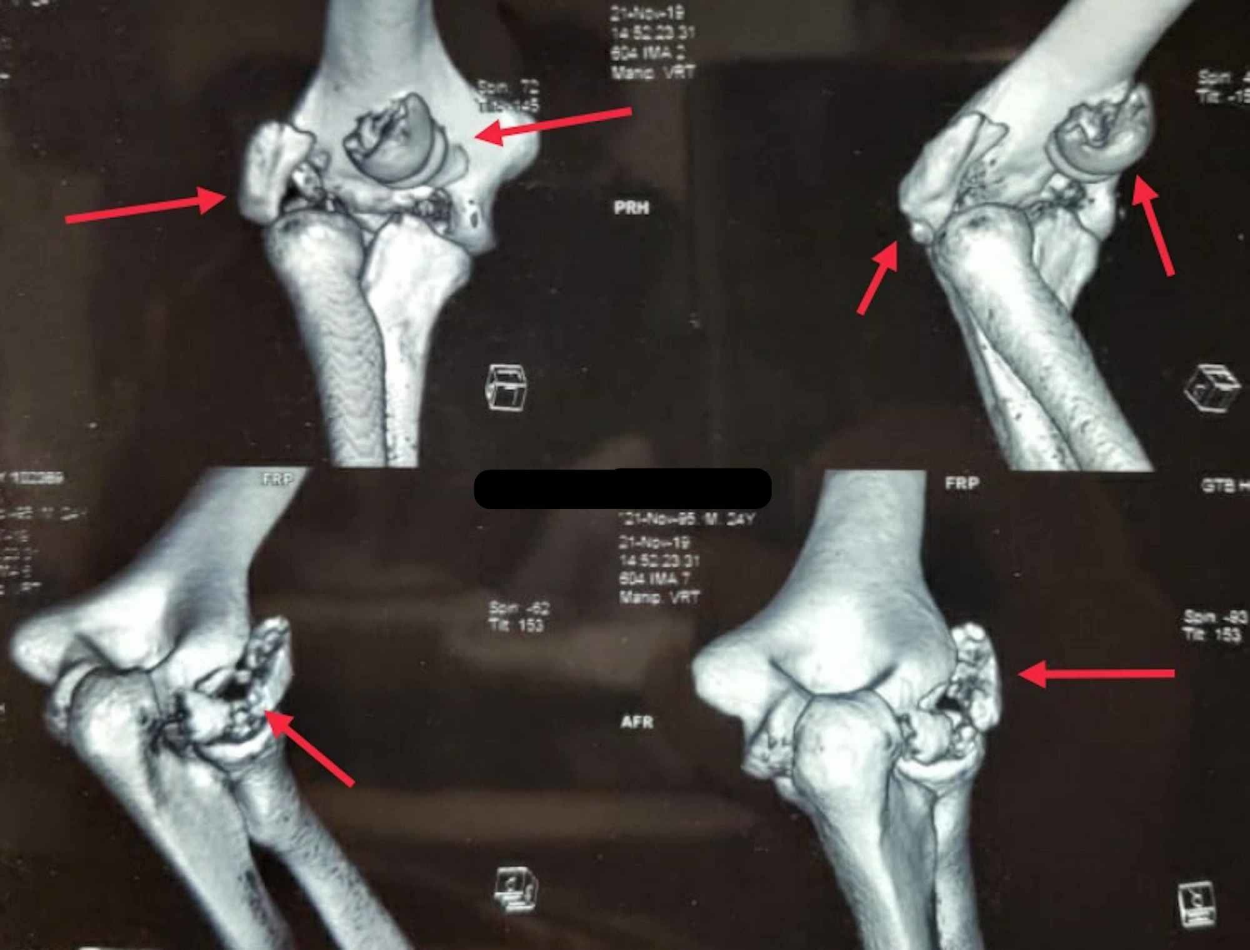
Three-dimensional reconstructed CT images of the right elbow. The arrows denote the capitellar fragments showing the exact fracture anatomy and comminution.

A CT scan of the elbow was done to delineate the fracture anatomy and it showed separate fragments of trochlea and capitellum with associated posterolateral comminution (Dubberley type 3b) (Figure 2). All blood investigations were normal. 

**Figure 3:**
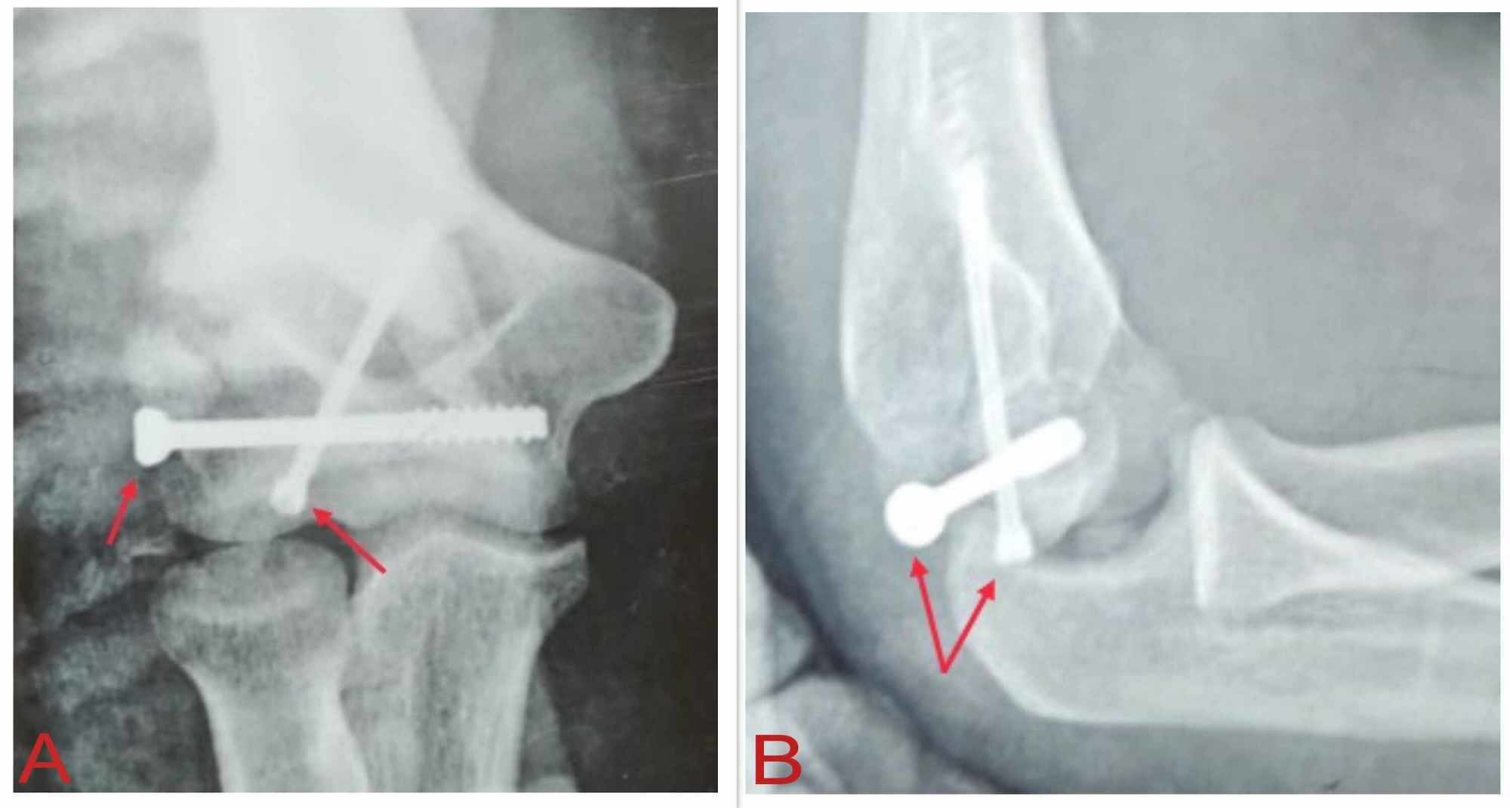
X-rays after open reduction and screw fixation of the capitellar fracture. The arrows denote the screws used for fixation in the (A) anteroposterior and (B) lateral views.

After taking informed consent, an open reduction and internal fixation of the fracture was done using the anterolateral approach to the elbow. Initial fixation of the anterior, large fragment was done with a single Herbert screw. After fixing the anterior fragment, a varus stress was given, along with traction, to open the joint, so that the posterior fragments could be pushed back into place. One large, lateral fragment was fixed with a lateral-to-medial cannulated cancellous screw (Figure 3). The wound was closed in layers over a negative-suction drain. No complication of wound dehiscence or neural deficit was observed postoperatively. A posterior above-elbow plaster slab was given for a week after which active, assisted elbow movements were started.

At six-month follow-up, the patient had no pain. He had a range of motion of 10-120 degrees in the flexion-extension arc, with full pronation and supination. Although he felt a slight loss of elbow strength, he was now able to do most of his daily activities without any difficulty. The Mayo Elbow Performance Index (MEPI) score was 92, suggesting an excellent result.

## Discussion

Capitellum forms the part of the distal humerus which articulates with the radial head. Fracture of the capitellum is an intra-articular fracture of the elbow, analogous to the Hoffa fracture of the knee. It is a rare fracture which represents an injury to the lateral column of the distal humerus. The mechanism of injury is usually an axial loading through the radial head [3]. 

The radiographic assessment has a major role. The CT scan in fracture of the capitellum is vital to know the exact fracture anatomy and to assess the amount of comminution. This helps the surgeon in determining the surgical approach and implant of choice. 

For coronal-shear capitellar fractures, most surgeons use the lateral approach for exposure of the elbow. The drawback of this approach is limited exposure of the medial articular extension and trochlea, making it difficult to recognize the actual fracture anatomy [4]. In our case, as the fracture line was extending medially to the trochlea, the anterolateral surgical approach was used as it provides adequate anterior exposure of the elbow joint. It also aided us in putting the anterior-to-posterior screw with ease. Although several screw types like cannulated, headless, cortical and cancellous have been used in fixation of capitellar fractures, studies have shown that Herbert screws provide adequate compression at the fracture site, and stable and anatomical fixation [5]. There is no intra-articular prominence of this implant.

Due to the propensity of the collateral ligaments and capsule towards contracture, the elbow joint is particularly vulnerable to postoperative stiffness [6]. Fracture of the capitellum being intra-articular further adds to this risk. Hence, early postoperative elbow mobilization is necessary for a good functional outcome. In our case, it was started on the seventh postoperative day.

## Conclusions

Comminuted fractures of the capitellum are extremely rare injuries and are difficult to manage. A preoperative CT scan should be done to assess the exact fracture anatomy and level of comminution. This helps the surgeon to decide the surgical approach and method of fracture fixation. The anterolateral approach to the elbow gives adequate anterior exposure and hence should be used in cases of capitellar fractures extending medially to the trochlea. A Herbert screw provides good compression at the fracture site and adequate stability with no intra-articular prominence. Early postoperative mobilization should be done to improve the functional outcome.
